# Can static Rorschach stimuli perceived as in motion affect corticospinal excitability?

**DOI:** 10.1371/journal.pone.0287866

**Published:** 2023-07-13

**Authors:** Agata Andò, Francesca Garbarini, Luciano Giromini, Adriana Salatino, Alessandro Zennaro, Raffaella Ricci, Carlotta Fossataro

**Affiliations:** 1 Department of Psychology, University of Turin, Turin, Italy; 2 Institute of Neuroscience (IoNS), Université Catholique de Louvain, Brussels, Belgium; Universita degli Studi di Trento, ITALY

## Abstract

It has been proposed that seeing human movement or activity (M), while trying to say what the static Rorschach inkblot design look like, is accompanied by Mirror Neuron System (MNS)-like mirroring activity in the brain. The present study aimed to investigate whether the Rorschach cards **eliciting** M responses could affect the excitability of the motor cortex by recording motor evoked potentials (MEPs) elicited by single-pulse TMS over the primary motor cortex (M1). We hypothesized that Rorschach inkblot stimuli triggering the viewer’s experience of human movement would increase corticospinal excitability. Twenty-one healthy volunteers (15 women) participated in the preliminary experiment, while another different sample of twenty-two healthy participants (11 women) ranging in age from 21 to 41 years was enrolled in the main experiment. Our results showed that the Rorschach cards known to be associated with a high number of M responses elicited human movement both as automatic internal sensations and as verbal production of responses involving human movement. However, contrary to our hypothesis, the reported internal feeling of human movement had no corresponding physiological counterpart, as the amplitude of MEPs did not increase. Possible and innovative explanations for the involvement of bottom-up and top-down processes were provided.

## Introduction

There is a widespread agreement in the motor literature that, regardless of the actual function of the Mirror Neuron System (MNS), the observation and execution of actions are not completely separate but are interdependent to some extent **(**e.g., [1]). Indeed, action perception influences action execution, and the latter in turn influences action perception [2]. Moreover, it is generally believed that the observation of a particular action/movement in conjunction with the embodied simulation of the same action is likely to trigger MNS-like activity [3]. Previous studies using transcranial magnetic stimulation (TMS) showed an increase in the motor system activity when dynamic information about bodily actions was derived not only from static pictures of postures [4], but also from abstract (static) figures and stimuli. More recently, a few electroencephalography (EEG) studies [5,6] investigated the activation of motor cortex for abstract artworks. Interestingly, the results of these studies showed that mu rhythm suppression can be elicited by passive observation of abstract art works (i.e., Lucio Fontana’s slashed canvases evoke the artist’s action to cut the canvas and look more dynamic with respect the control stimuli); thus, the cortical motor system appears to be involved in the viewing of static abstract art works. As well as for the actions observed, perceived, performed, and imitated, the involvement of the cortical motor system also depends on the interaction with emotional stimuli. Emotions are thought to be closely associated with actions and often trigger and automatically guide our behavior for interactions. For example, in an early phase (i.e., 150 ms) an enhancement of corticospinal excitability for the observation of happy and fearful emotional faces compared to neutral expressions was observed specifically in the right hemisphere; interindividual differences in the disposition to experience negative emotions in interpersonal emotional contexts also predicted the early increase in corticospinal excitability to emotional facial expressions [7]. Viewing people in emotional pictures (i.e., IAPS stimuli) increased motor excitability compared to viewing landscapes or household objects [8], suggesting that emotional cues can stimulate the body to act [9].

### The ambiguous, Rorschach stimuli and the spontaneous attribution of human movement

The Rorschach test consists of 10 ambiguous inkblot designs. Typically, subjects are asked to tell the examiner what they see in the inkblots. Their responses are then interpreted based on (a) what they see, (b) what they saw in the inkblot (e.g., the shape, the color, etc.), and (c) where in the inkblot they saw each of their perceptions. According to various Rorschach theorists [10,11], the human movement responses (M) indicate the ability to use one’s imagination to elaborate human experiences or activities, ideation, interpersonal cognition, and related constructs such as imagination, intelligence, reasoning, and thinking before doing. In other words, a person who sees, for example, “a man lifting an object that is much too heavy for him” is thought to implicitly identify himself to some extent with the character of the response, thereby revealing important information about him/her-self.

Several data support the validity of the M response in the context of an *identification* mechanism [12–16]; the psychological process that triggers M responses is similar to theoretical constructs such as Einfühlung (a German word literally meaning "feeling-in" which has been adopted into the experience of art referring to the immediate physical responses generated in the viewer by the exposure to art painting), empathy (the ability to understand the sensations of others, for example), and also mentalization (e.g., [17–19].

According to the idea that the M depends on a mechanism of identification or embodiment, it was proposed that the spontaneous association of M with ambiguous or partially unstructured visual stimuli (such as some Rorschach inkblots; i.e., II, III, VII card; [20]) would be accompanied by MNS-like mirroring activity in the brain. To date, this hypothesis has been supported by EEG [21] and repetitive TMS (rTMS) [14,15] studies. In a first study [21], EEG data were collected while participants were exposed to a set of four Rorschach inkblots and pictures that were similarly designed. Given that the suppression of the 8–13 Hz wave at C3, Cz, and C4 scalp locations (i.e., mu suppression) is thought to be an index of mirroring activity in the brain [22,23], the authors hypothesized that attribution, identification, and observation of human movement would be associated with increased EEG mu suppression, compared with control conditions. The results supported this hypothesis, leading to the conclusion that the internal representation of the “feeling of movement” might be sufficient to trigger MNS activity even in the presence of minimal external cues. Subsequent EEG studies [24,25] examined all ten Rorschach cards in a larger sample confirming similar effects to the previous ones. More recently, an rTMS study provided additional data supporting the relationship between M responses, embodied simulation, and mirroring activity in the brain. Andò et al. [14] administered a subset of Rorschach inkblots to a sample of non-clinical adults during a baseline condition (without rTMS) and soon after inhibitory rTMS. Half of the participants (i.e., the experimental group) were stimulated over the Left Inferior Frontal Gyrus (LIFG, a putative MNS-like activity area), whereas the other half (i.e., the control group) were stimulated over the Vertex (a control area). Consistent with the hypothesis that the generation of M responses is associated with mirroring activity in the brain, the LIFG disruption, but not the Vertex, resulted in a statistically significant reduction in the tendency to perceive human movements in the ambiguous Rorschach inkblots compared with the control condition. These results were replicated in a second rTMS study [15] in which EEG data were recorded: disrupting the LIFG but not Vertex, decreased the number of M attributions but unexpectedly rTMS did not significantly influence EEG activity.

Finally, a more recent fMRI study [26] reported that M responses were associated with embodied simulation and MNS**-**like activity: univariate region-of-interest analyses showed that production of M responses were associated with significantly higher activity in MNS-related brain regions when compared to non-M Rorschach responses. Overall, all of the above results were consistent with the traditional interpretation of the M response**s** as an embodied simulation process, feeling of movement, and empathy components.

Against this background, the present study aimed at investigating whether the Rorschach cards evoking M responses could affect the motor-cortex excitability as measured by motor evoked potentials (MEPs) induced by single pulse TMS over the primary motor cortex (M1). We hypothesized to observe an increase in corticospinal excitability when exposed to Rorschach cards closely associated with M response.

## Method

### Participants

The study consisted of two separate experiments, i.e., the *preliminary* and the *main* experiment. Twenty-one non-clinical healthy volunteers (15 females) ranging in age from 22 to 26 years (*M* = 23.95; *SD* = 1.20) participated in the preliminary experiment whereas a different sample of twenty-two healthy participants (11 females) ranging in age from 21 to 41years (*M* = 25.32; *SD* = 4.03) was enrolled in the main experiment. All participants were right-handed, as assessed with the *Edinburgh Handedness Inventory* [27], naïve to the experimental procedure and before taking part in the study gave written informed consent. None of them had a history of neurological, major medical, or psychiatric disorders and participants enrolled in the main experiments were free from any contraindication to TMS [28] based on self-reported information and TMS screening checklist. The experimental procedure, according to the Declaration of Helsinki, was approved by local Ethics Committee of the University of Turin (number of protocol: 3167)

### Rorschach performance assessment system (R-PAS)

The Rorschach Performance Assessment System (R-PAS; [13]) is a relatively new, psychometrically sound, evidence-based Rorschach method developed to overcome most of the limitations of previous systems. R-PAS has so far proven to be a reliable [29–31] and valid (e.g., [14,32–34] method for administering, coding, and interpreting the Rorschach. As a performance-based personality test, the basis for Rorschach interpretations rests on the fact that behaviors observed in the microcosm of the task (i.e., while taking the Rorschach test) are likely to generalize to behaviors occurring in the outside world. Thus, people who tend to be oppositional and aggressive in the Rorschach test are likely to exhibit these traits outside the test situation as well (i.e., in their daily lives) [13]. The administration consists of two phases: The *Response Phase* (RP), in which the examiner asks the respondent to answer the question, *“What might this be*?*”*, and the *Clarification Phase* (CP), in which the examiner asks a series of questions to “clarify” the coding and resolve ambiguities.

### The preliminary experiment

The preliminary experiment was performed in order to verify whether the administration of the Rorschach’s inkblot cards on a PC display was effective in inducing a feeling of movement in the observer as the standard administration. To this aim, a total of six Rorschach cards were selected, three cards (i.e., *M Cards*; II, III and VII) with the highest frequency of human movement attribution (M responses), and other three cards (i.e., *Non-M Cards*; V, VI and VIII) with the lowest frequency of human movement [13,20]. The preliminary experiment consisted of two experimental sessions differing for the Rorschach’s cards presentation. In the first one, participants underwent to the Rorschach’s cards presentation at the PC screen (PC administration), while in the second one the Rorschach’s cards presentation followed the standard procedure (standard administration), see details below. This allowed to directly test if the two types of Rorschach administration are equivalent in inducing the perception of feeling of movements.

In the “PC administration”, stimuli were presented on PC screen (17” monitor; resolution 1280x720 pixels; refresh frequency 60 Hz) placed at a distance of ~80 cm away from the participants. The experiment was programmed by using E-prime presentation software V2.0 (Psychology Software Tool Inc., USA) in order to a) control stimulus’ sequence, timing and duration and b) record the participants’ responses. The experiment consisted of a total of 30 stimuli presented in a pseudorandom order, 15 of them were M cards (5 II, 5 III, and 5 VII) and the other 15 were Non-M Cards (5 V, 5 VI and 5 VIII). Each card was presented for 300 ms and then participants were asked to judge the amount of the perceived feeling of human movement (i.e., *“how much human movement do you perceive in the presented inkblot*?”) using a 9-points Likert scale ranging from 0 “not at all” to 9 “definitely” (the participants verbally gave their number so as not to make hand movements; after listening to the number, the experimenter entered the value in E-prime). A black screen with a fixation cross with a variable jittering (12000–16000 ms) was presented before the next trial. In this study, we used the stimulus duration of 300 ms since this timing resulted effective for an increase in (hand) motor excitability in previous TMS studies (e.g., [8,35–37]) showing that neural activity reflecting motor resonance is typically detected at about 250–350 ms after stimulus onset in the motor cortices.

In the “standard administration” session (performed after the exposure of the Rorschach stimuli during the “PC administration”), the Rorschach test was administered according to R-PAS method by selecting only M and Non-M cards, as follows (see also, S1 Fig).

*Scenario 1* –*Response phase (RP)*: *The respondent immediately remembers and reports the object seen during the “PC administration”*. In this case, the examiner shows the first card and asks: *“Concentrate*, *please*. *Do you remember what you saw before*, *in this card*?*”* If yes, examiner says: *“What might this be*?*”* If few responses emerge during the RP e.g., only one response per card the examiner goes through the cards again in the correct order (from Card II to Card VIII) and encourages the respondent to give a few more answers: *“However*, *we need a few more responses for the test to be helpful*. *So let’s go through the cards again*. *Take your time when looking at them and see what other things you can come up with”*. Examiner hands the respondent the first card and says: *“What else might this be*?”

*Clarification Phase* (CP): Examiner follows the standard R-PAS-CP. The Clarification Phase exists only to obtain data for accurate coding of what was seen during the Response Phase. The standard manner to introduce the Clarification Phase is as follows: “*Now we are going to start the final step*. *While looking at the cards I want to review your responses with you to clarify what it is that you saw and how you saw it*. *So we will look at the cards one by one*. *I will read your responses back to you and I want to know where on the card you were looking and what about the inkblot made it look like that to you*. *Does that make sense*?*”*

*Scenario 2—RP*: *The respondent” does not remember having seen any specific object of “PC administration*, *when examiner shows the first card*. In this case, examiner follows the standard R-PAS administration: *“What might this be*?*”*

*CP*: Examiner follows the standard R-PAS-CP.

*Scenario 3—RP*: *The respondent does not remember having seen any object when the same Rorschach cards are shown*, *but during the exposure to the Rorschach test [T2] they remember their previous responses (provided during the [T1] “PC administration”)*. In this case, examiner follows the standard R–PAS administration.

*CP*: Examiner follows the standard R-PAS-CP.

In the current study, two independent raters coded for the presence vs. absence of M responses. The percentage of agreement was 98%, the ICC was 0.98.

#### The Main Experiment

The main experiment aimed to investigate whether the corticospinal excitability could be affected by the Rorschach inkblot cards, being some of them able to induce a feeling of movement in the participants and others not [20]. To this aim, as in the preliminary experiment, participants underwent both the “PC administration” and the “standard administration” of the selected Rorschach’s inkblot cards.

**Fig 1:**
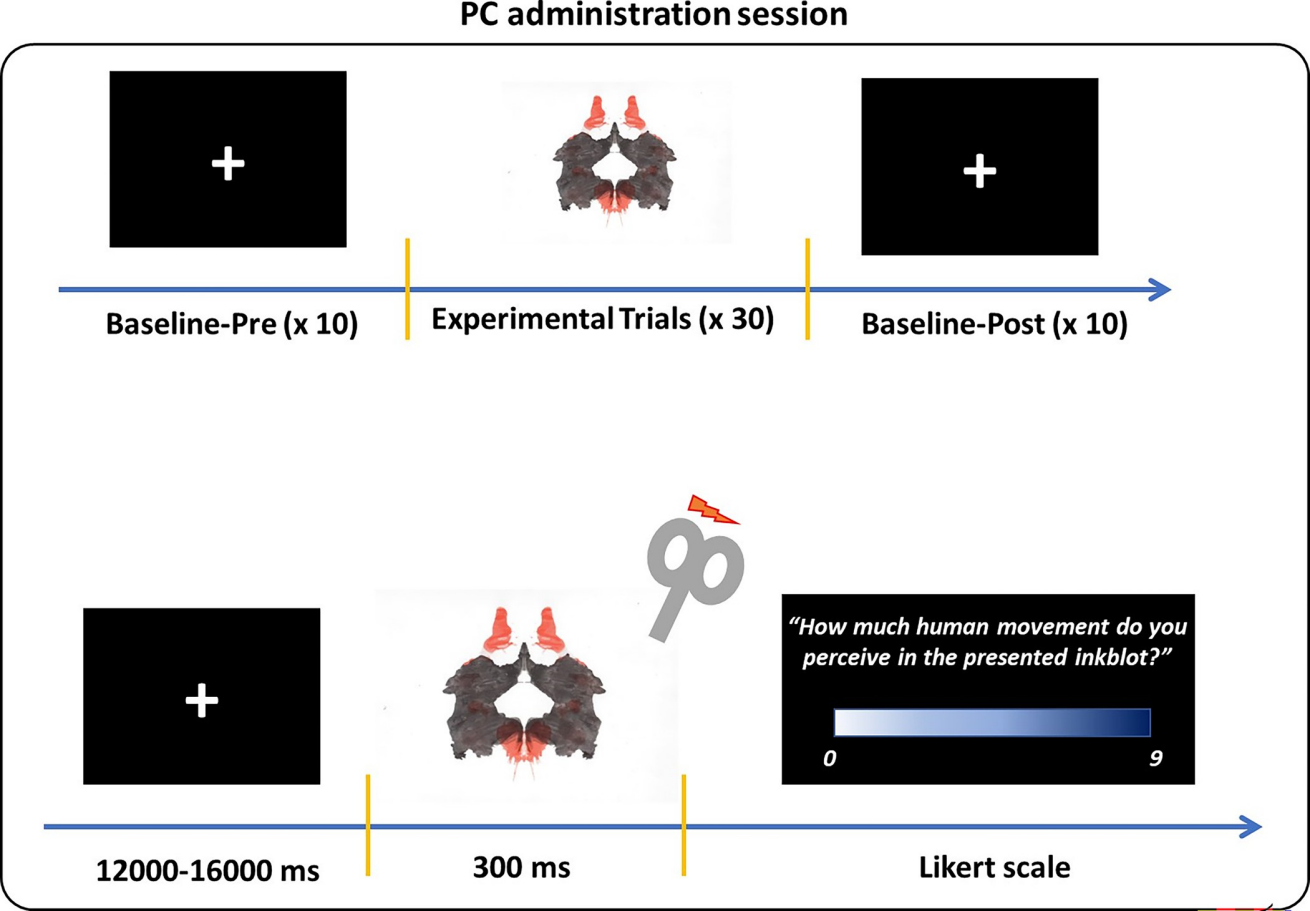
Experimental administration session. *Top panel*, schematic representation of the TMS protocol timeline. *Bottom panel*, schematic representation of a single trial.

The “P he psychological process that triggers MC administration” session (see Fig 1), as in the preliminary experiment, consisted of a total of 30 stimuli (15 M card and 15 Non-M card) presented in a pseudorandom order by an E-prime (V2.0 Psychology Software Tool Inc., USA) for 300 ms with a jittered interstimulus interval of 12000–16000 ms. Note that the number of trials was based on the trials number of previous TMS experiment (see for e.g., [38–41]). According to previous studies [35–37] showing that neural activity reflecting motor resonance is typically detected at about 250–350 ms after stimulus onset in the motor cortices, the TMS pulse over the primary motor cortex (M1) was delivered at the off-set of the card presentation (i.e., at 300 ms) and MEPs were recorded. Then, as in the preliminary experiment, the amount of the perceived feeling of movement was measured through a 9-points liker scale appearing on the screen. Baseline measures of the corticospinal excitability were also assessed before (i.e., baseline pre) and after (i.e., baseline post) the experimental block by means of two supplementary series of 10 MEPs. A fixation cross lasting 10050 ms was presented in the centre of the screen and TMS pulse was delivered 10000 ms after the fixation cross onset. These series of MEPs were used to check for any corticospinal excitability change related to TMS per se between the beginning and the end of the experimental block and their average amplitudes were calculated to set individual baselines for data normalization (see details in data analysis section). Participants were seated comfortable in front of a PC screen (17” monitor; resolution 1280x720 pixels; refresh frequency 60 Hz) at a distance of ~80 cm, with the head restrained by a comfortable pillow wrapping around the neck and supported by a fixed head rest; in order to avoid any muscles contractions, they were asked to keep resting their forearms on a pillow.

In the “standard administration” session (after the exposure of the Rorschach stimuli during the “PC administration”), the Rorschach test was administered according to R-PAS method, but by selecting M and Non-M cards only (in the same way reported in the preliminary study; please, see the preliminary study section above for details).

#### Transcranial magnetic stimulation and electromyography recording

MEPs were elicited by single pulse TMS trough a biphasic stimulator (Magstim Rapid^2^; Magstim Co. Ltd, Whitland, UK) with a figure-of-eight-shaped coil (D70 Alpha flat coil) positioned over the left M1 (hand area). The coil was held tangentially to the scalp with the handle pointing backwards and laterally 45° away from the mid-sagittal line, such that the flow induced by the second most effective phase of the biphasic pulse moved in an anterior-posterior direction, thus behaving more similarly to a monophasic posterior-anterior pulse[42,43]. order to determine the optimal position able to elicit the greatest amplitude MEP with the lowest stimulation intensity, this orientation permits the lowest motor threshold, optimizing the stimulation [44]. By moving the coil in step of 1 cm over the left motor cortex the optimal point able to activate the selected muscle was found, then the coil was fixed and held by a mechanical arm. The intensity of magnetic pulses was set at 115% of the resting motor threshold (Mean±SD 64.56%±9.81%, range 48.3–80.5% of the maximum stimulator output), defined as the lower intensity of the stimulator output able to elicit five MEPs of ten consecutive pulses with an amplitude of at least 50 μV [45].

Electromyography (EMG) activity was simultaneously recorded (MP150, Biopac System, USA), from the right Abductor Pollicis Brevis (APB) and the right First Dorsal Interosseous (FDI) muscles, using two pairs of bipolar surface electrodes with the active electrode over the muscle belly and the reference electrode over the associated joint or tendon. Signals were amplified and digitalized with a sample rate of 10 kHz, filtered with a band-pass (10–500 Hz) and a notch (50 Hz), according to the method used in previous studies [39–41,46–51], and stored for off line analysis.

### Data analysis

#### Behavioral data

In both experiments, the participants’ ratings about the perceived feeling of movement were analyzsed by means of paired t-tests (two-tailed). To detect violations of normality, we checked the distribution of residuals by Shapiro-Wilk’s test (p>0.05). Note that we did not use nonparametric statistics because the VAS (Likert) scale did not depart substantially from normality (i.e., skewness < 2 and kurtosis < 7; [52]).

### MEPs analysis

EMG data were analysed offline using AcqKnowledge software (Biopac Systems, Inc., Santa Barbara, CA). In order to prevent contamination of MEPs by background EMG activity, the absence of any voluntary contraction in 100 ms window preceding the TMS pulse was verified by visual inspection monitoring the EMG activity online. For each muscle, all trials with any activity greater than 50 μV were excluded from the analysis (less than 2% of collected data [41,48]). For each participant and separately for each experimental condition (i.e., M cards; Non-M cards) the average peak-to-peak MEPs’ amplitude (μV) was extracted and used as dependent variable for further analyses. First, to control for the possible effect of TMS per se in modulating corticospinal excitability, a preliminary analysis was conducted on the baseline mean raw MEP values by means of paired t-tests (two-tailed), separately for each muscle. In the main analysis, the MEP values recorded during the experimental block were averaged and expressed as percentage of the mean MEP values recorded from the baseline (MEP ratio = MEP_experimental_/MEP_baseline_), and then a natural log(x+1) transformation [53] was applied to the raw data. The normalized data were analysed by means of paired t-tests (two-tailed), separately for each muscle. To detect violations of normality, we checked the distribution of residuals by Shapiro-Wilk’s test (p>0.05).

### Correlation analysis

Correlations were performed to examine whether a relationship existed between a) the behavioural data [i.e., feeling of movement ratings during the exposition to the M Rorschach cards and to the Non-M Rorschach cards] and verbally production of M responses to Rorschach test (R-PAS), in the preliminary experiment; and b) the physiological data (i.e., MEP values in the high M frequency Rorschach cards and Low M frequency Rorschach cards), behavioral data (i.e., feeling of movement ratings during the exposition to the M cards and **Non**-M Rorschach cards) and verbally production of M responses to Rorschach test (R-PAS), in the main experiment. A linear regression analysis was performed and the normalized MEP values were used to predict the participants’ ratings.

## Results

### The preliminary experiment

T-test performed on the participants ratings showed a significant difference between the M cards and the Non-M Cards [*t*_*(21)*_ = -3.29; p = 0.003], suggesting a significantly greater perceived feeling of movement at the M cards (Mean±SD 3.61± 2.63) as compared to the Non-M Cards (Mean±SD = 1.97±1.70). We found marginally statistically significant correlations (*r* = 0.428; *p* = 0.053) between perceived feeling of human movement at the M cards in E-Prime PC administration (i.e., subjective ratings) and the M responses at the M Rorschach cards, in R-PAS standard administration. These results confirmed that the experimental manipulation had the expected effect, so we proceeded with the main experiment, which also included the recording of MEPs.

### The main experiment

The t-test performed over the participant’s ratings confirmed the results obtained in the preliminary experiment (Table 1). We observed a significant difference between the M Rorschach cards and Non-M Rorschach cards [*t*(*21)* = 4.93; *p* ≤ .001 *d* = .05], suggesting a significantly greater perceived feeling of human movement at the M cards (M = 3.80; SD = 2.28) as compared to the Non-M Cards (*M* = 1.70; *SD* = 1.62). Furthermore, we observed statistically significant differences in the verbal production of M responses to the Rorschach test between the high M frequency Rorschach cards and Low M frequency Rorschach cards (*t*(*21)* = 6.30; *p*≤.001; *d* = 1.34] suggesting a significantly greater number of M responses elicited by the associated type of Rorschach Cards (M cards, *M* = 3.05; *SD* = 1.50; Non-M cards, *M* = 0.68; *SD* = 1.62). The t-tests performed on the MEPs data collected during baseline did not show any significant difference (*p* > .05; Table 1), thus ruling out any effect of the TMS per se in modulating the corticospinal excitability. The t-tests performed on the MEPs data (log transformed) did not show any significant difference between the M and Non-M Cards neither in FDI (*t*(*21)* = -.35; *p* = .73) nor in APB muscle (*t*(*21)* = .39; *p* = .70).

**Table 1:** Comparison between M and Non-M stimuli.

	M Cards		Non-M Cards		t_*(21)*_	*p*	*d*
	*Mean*	*SD*	*Mean*	*SD*			
VAS	3.80	2.28	1.70	1.62	4.93	< .001	1.05
Rorschach M	3.05	1.50	.68	1.17	6.30	< .001	1.34
MEP FDI (log) Raw data (mV)	-.066 .37 mV	.239 .28 mV	-.058 .4 mV	.209 .33 mV	-.35	.73	-.07
MEP APB (log) Raw data (mV)	.043 .34 mV	.294 .21 mV	.029 .35 mV	.202 .22 mV	.39	.70	.08

*Note*: VAS = perceived feeling of human movement measured by VAS; Rorschach M = verbal production of M responses reported during R-PAS standard administration.

We next conducted correlations between all variables, i.e., feeling of human movement (i.e., Average of VAS), verbal production of M responses to the Rorschach (i.e., Average of Rorschach M), physiological data (i.e., Average of MEP FDI and Average of MEP APB) (Table 2). We did not find any significant correlations between behavioral and physiological data. Also, surprisingly, the M responses to the Rorschach test correlated in the expected direction but not significantly with VAS, in M (Rorschach) cards (*r* = .115; *p* = .609) and Non-M cards (*r* = .165; *p* = .454).

**Table 2:** Correlation between VAS, Rorschach M and MEP.

	Avg VAS	Avg Rorschach M	Avg MEP FDI	Avg MEP APB
M Cards				
Avg VAS	1.00	.115	-0.276	-0.269
Avg Rorschach M	.115	1.00	-0.095	-0.129
Avg MEP FDI	-.276	-.095	1.00	.517*
Avg MEP APB	-.269	-.129	.517*	1.00
Non-M Cards				
Avg VAS	1.00	.165	-.085	.023
Avg Rorschach M	.165	1.00	.073	.152
Avg MEP FDI	-.085	.073	1.00	.469*
Avg MEP APB	.023	.152	.469*	1.00
All (six) Cards				
Avg VAS	1.00	.141	-.237	-.137
Avg Rorschach M	.141	1.00	.01	.63
Avg MEP FDI	-.237	.01	1.00	.510*
Avg MEP APB	-.137	.063	.501*	1.00

*p ≤ .05

** p ≤.01

*Note*: Avg VAS = Average of perceived feeling of human movement measured by VAS; Avg Rorschach M = Average of verbal production of M responses reported during R-PAS standard administration.

## Discussion

Our results are partially in the expected direction. Indeed, it is possible to observe how the II, III, and VII Rorschach cards evoked human movement both as an automatic-internal *sensation* and verbal production of responses involving human movement, compared to the V, VI and VIII Rorschach cards. We can therefore assume that during the exposure to the M Rorschach cards the observer experienced a “feeling of movement” within their body. This finding is in line with the idea that the Rorschach inkblot stimuli are related to those processes underlying the ‘‘feeling of movement” in situations in which a person is wondered to understand, elaborate, and/or attribute meaning to ambiguous stimuli. However, we found no significant statistical correlations between the feeling of human movement reported by subjective ratings (i.e., at the PC administration) and the verbal production of the movement-inclusive response (i.e., at the standard administration). Furthermore, the reported internal feeling of human movement had no corresponding physiological counterpart, as the amplitude of MEPs did not increase. Possible explanations for these results are discussed below.

### No correlations between VAS and Rorschach M responses

#### The feeling of the implied and inner movement may not be reflected in its conscious representation and verbalization: The role of bottom-up and top down processes

An unexpected finding was the lack of statistically significant correlations between the VAS, and thus how strongly a participant perceived the sensation of human movement during the PC administration, and the production of human movement responses to the Rorschach test. As mentioned above, this result was unexpected given that previous studies have shown that exposure to some Rorschach card elicit a feeling of movement [14,21,25], as (self) reported by subjects and also at the neurophysiological level. According to complex models of attentional processing [54–57] perception is the results of both bottom-up and top-down processes. Thus, when one looks at the inkblots and provides responses, two attentional networks would be active, the ventral attention system that automatically processes stimulus information related to object properties such as shape and color, and the dorsal attention system [58], which is typically involved in top-down processes allocating the attention “voluntarily” toward specific locations or features [26,59]. Hence, during the Rorschach test, we can assume that firstly the automatic and visceral bottom-up process (i.e., emotional and linked to body sensitivity) takes place starting from the sensory input provided by the exposition to the Rorschach card. Then the top-down process involving the dorsal system takes place, reflecting the fact that a person trying to find their answer to the question “what might this be?” is likely to actively scan some areas of the inkblot, searching for the best locations within the card, to give their answer. The more ambiguous is the represented object the more extensive is the required mental processing. This voluntary and most *aware* phase of the process occurs and the incoming data are selected and filtered, undergoing encoding and decoding processes, and are adapted and readapted to the cognitive schemes already present. We can speculate that there is a substantial difference between what a person feels internally when exposed to the ambiguous, undefined stimulus (which escapes awareness) (i.e., the bottom-up process) and what they perceive consciously instead, having adapted some aspects of the visual feature of the ambiguous stimulus to previous cognitive schemas (i.e., the top-down process). Therefore, the M-Rorschach cards may be related to existence of *different pathways* (underlying two different mechanisms that interact between them): a) an internal perception of the human movement from the sensory data (e.g., parts of a stimulus) (*bottom-up* process) and b) the continuous adjustments and readjustments to stored information during the standard administration (*top-down* process) leading to the verbal production of M-answers (especially during the R-PAS response Phase; e.g., [60]). Indeed, it has been reported that a great deal of dorsal attention network activity is involved in the verbal “construction” and production of M responses [61,62].

### No relationship between feeling of movement and MEPs

#### Feeling of movement and motor facilitation: Is there a relationship?

Activation of the motor cortex for abstract artworks was studied using EEG [5] and ERPs [6]: engaging the viewer’s motor system facilitates simulation of the sensorimotor correlates of the actions depicted on a canvas and/or the actions of the artist in producing an art-work (e.g., the actions/brush strokes required to produce a painting or sculpture; [63]. This motor simulation is associated to an empathic response to a work and ultimately contributes to its aesthetic appreciation [64,65]. Consistent with this view, single-pulse TMS [66] and neuroimaging studies (e.g., [67]) have shown greater activation of fronto-parietal areas known to link action performance to action observation [1] during viewing of paintings compared to modified non-artistic stimuli. Previous studies [4,8,68] reported that the action simulation promotes corticospinal excitability mainly in the muscles used in the execution of the observed movements, occurring approximately 200 ms after stimulus presentation [68–70]. Therefore, the motor activation specifically reflects the simulation of the motor aspects of the artist’s painting acts and would necessarily require the involvement of the affected body district. Furthermore, several studies have provided evidence for even stronger motor activation in response to actions that are further not easily available in and comparable with the observer’s motor repertoire [71]. In all these cases, motor activation does not seem to reflect ease of simulation, but rather an attempt to cope with unusual or completely new movements using familiar motor representations.

The Rorschach cards “do not include” *defined* or *already performed* movements: by a mechanism of identification the subject *adds* to the inkblots the (human) movement, a characteristic that a Rorschach inkblot “intrinsically” does not have. Differently from Fontana paintings or the brushstrokes on canvas in which the observer can identify exactly a movement of the arm/wrist extensor parts, in the Rorschach ambiguous/not defined stimuli a movement is interpreted and then attributed [13,34]. Therefore, we can speculate that the observer—just immediately exposed to the Rorschach card—does not necessarily have to draw massively from their motor repertoire (see previous paragraph and the bottom-up process): all this could trigger some kind of automatic embodiment without mediation of increased motor activity and/or corticospinal excitability. This assumption is consistent with Finisguerra’s findings [72] showing that lower motor activation was present in those subjects who reported a higher willingness to adopt the cognitive perspective of others and embodied simulation. The latter result seems to be consistent with the traditional interpretation of the feeling of movement related to M responses generated by exposure to the Rorschach cards (e.g., [14,73]) which is probably not necessarily associated with the activity of the M1 area and the resulting increase in the amplitude of the MEPs. A recently proposed model for action observation (Dimensional Overlap Model) outlining the evolving dynamics of the motor system during action observation in order to react to it following rule-based visuomotor matching, assumes two independent parallel routes, an automatic and a rule-based one. Thus, according to this model the automatic activation of motor representations (motor resonance) is not an obligatory step for applying rule-based visuo-motor transformations [70].

There is a significant difference between what a person feels internally when exposed to the ambiguous, undefined stimulus (bottom-up process) and what they elaborate consciously instead after adapting some aspects of the visual features of the ambiguous stimulus to previous own cognitive schemas (top-down process). Therefore, we can hypothesize that only prolonged exposure to the Rorschach cards (e.g., [21,74]) and continuous adjustments and readjustments of the stored information during the standard administration (related to the top-down process; e.g., [61]) leading to verbal production of M responses could possibly increase the cortical excitability and thus the greater activity of M1 and the consequent increase in the amplitude of MEPs. In summary, the sensation of movement may also occur immediately—this would be consistent with the analysis of timing in Pineda et al. [25] where the suppression of M was observed as early as the first few seconds. However, the generation of M probably also requires cognitive and verbal activity (and this is consistent with the various Rorschach theories contrasting M and color, stating that the former is highly cognitive and the latter more spontaneous). In support of this extra cognitive effort to give M responses, it is possible to note that in Ales et al. [62] M responses were indeed associated with longer-lasting visual fixations than variables likely to reflect more spontaneous behaviors (e.g., Vg% or color). Thus, given the intrinsic ambiguous nature of the Rorschach inkblot, the lack of any modulation effect on the corticospinal excitability can be reasonably ascribed to the adopted timing of the TMS pulse which could be too short. Indeed—in contrast to previous results reported observing static real human body [8] finding a motor system activations after ~300 ms—a recent study by Fiori and colleagues [75] investigated corticospinal excitability while participants observed paintings depicting either human and non-human scenes with either static or dynamic posture/scenes. They observed that only paintings conveying the impression of a dynamic human movements enhanced the amplitude of MEPs. However, in this study the TMS pulse was delivered 600 ms after a painting was presented. Battaglia and colleagues collected MEPs after 3 seconds while participants were asked to observe the Michelangelo’s Expulsion from Paradise painting; similarly, Umiltà and colleagues [5] reported mu rhythm suppression in a time window ranging from 250 to 750 ms after the appearance of the original art works. Rorschach inkblots are abstract stimuli that do not convey motion-related information *per se*, thus participants’ corticospinal system probably would have need more time to “resonance” with such stimuli. Hence, we may supposed that if we had increased the timing between the stimulus presentation and the TMS pulse, it would have been more likely to detect corticospinal modulation.

Furthermore, in the present study, we focused only on the hand muscles, assuming that any feeling of movement induce by the observation of the card would have involved a hand movement. However, the perceived movement would not necessarily imply hand muscles. Hence, another possible explanation of the lack of corticospinal-excitability modulation could be that participants might have re-constructed a human movement involving other body districts (e.g., upper arm, leg or face).

**Fig 2:**
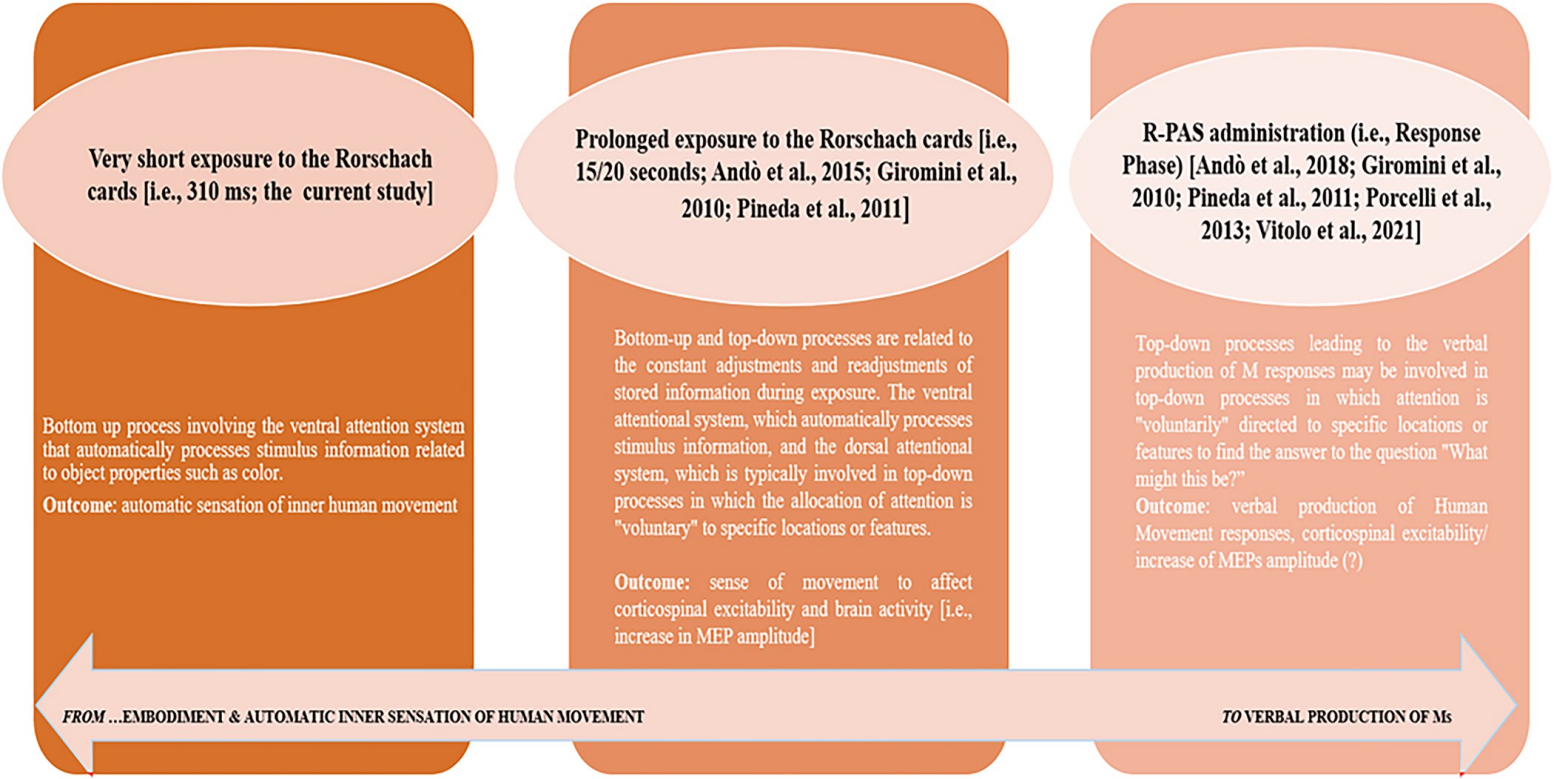
From embodiment to Ms verbal production (R-PAS; 2011): Findings and hypotheses.

Our considerations described above are shown graphically below (see Fig 2).

## Conclusion

The results obtained are only partially in the expected direction. Nevertheless, it is necessary to emphasize the relevance of the information obtained from the study and to show how the aspects of motor activity are related to those of testing. The study is characterized by techniques and a general methodology anchored in previous studies and results already validated in the literature. The idea that testing exposure involves both bottom-up and top-down processes is noteworthy [32]: there could be a first phase in which an automatic and visceral bottom-up process starts from the sensory input provided by the exposure to the Rorschach stimulus, and a second phase in which a top-down process takes place, in which attention is “voluntarily” directed to specific locations or features of the Rorschach cards. In this second and most deliberate phase of the process, the incoming data are selected and filtered, undergoing encoding and decoding processes and being adapted and readapted to the cognitive schemas already in place. During the Rorschach test, an individual may be involved by automatic, implicit elements that escape consciousness but affect the production of the human movement/observable behavioral response. The production of the human movement response could mark a subsequent phase of voluntary readjustment, which may be accompanied by an increase in the amplitude of MEPs and cortical excitability/activity (see, e.g., [21,61]) that occurs after a temporally prolonged exposure to the cards and is then manifested in the concrete verbalization of a response in which human movement is present; an observer who is directly exposed to the Rorschach card does not necessarily have to draw massively from his motor repertoire but the automatic embodiment may occur without mediation of increased motor activity and/or corticospinal excitability.

What has just been described may seem to contradict previous studies on the Ms on the R-PAS [15,25,26], but it should be underlined that in these studies, participants were exposed to the Rorschach cards for more seconds (from 10 to 30 seconds): therefore, due to the very short exposure time of 300 ms, probably no correlation was found in our study between the VAS—which was used to evaluate the sensation of movement—and the elaborated and constructed human movement (verbal).

### Limitations

This study has obvious limitations: the small sample, the fact that MEPs were detected in APB and FDI muscles and not in other parts of the body that might be involved and activated during exposure to the Rorschach cards, and no detection of MEPs over an extended period of time (and/or specifically when the subject gave the type of response during standard R-PAS administration although this would have caused several artifacts). In addition, the ecological validity of this study is very limited: the R-PAS Rorschach test is administered in a different way, e.g., by showing the 10 cards and conducting the RP and the CP in a quiet place without the interference of machines and other factors; previous studies used the same timing, but the stimuli to which participants were exposed, even if static and/or ambiguous, differed from the Rorschach cards. Finally, we also attempted to find an explanation for the expected and unexpected results by including information from previous studies, but we did not measure dorsal vs ventral activity in this study.

However, our study has the merit of being the first and only study to investigate the excitability of the cortex based on the amplitude of MEPs during the strong visual stimuli of the Rorschach test. How does the sense of movement emerge and develop over time? When does embodiment transition to verbal production of the human movement response, index of higher cognitive functions related to social cognition and empathy. The study attempted to provide answers to these questions by reporting considerations and hypotheses.

## Supporting information

S1 Fig Standard R-PAS administration procedure. (PDF)

S2 Fig Correlation Graph. (PDF)

## References

[1] Rizzolatti G, Craighero L. The mirror neuron system. Annu Rev Neurosci. 2004;27: 169–192. doi: 10.1146/annurev.neuro.27.070203.144230 15217330

[2] Witt JK. Tool use influences perceived shape and perceived parallelism, which serve as indirect measures of perceived distance. J Exp Psychol Hum Percept Perform. 2011;37: 1148–1156. doi: 10.1037/a0021933 21500947

[3] Gallese V, Sinigaglia C. What is so special about embodied simulation? Trends Cogn Sci. 2011;15: 512–519. doi: 10.1016/j.tics.2011.09.003 21983148

[4] Urgesi C, Moro V, Candidi M, Aglioti SM. Mapping implied body actions in the human motor system. Journal of Neuroscience. 2006;26: 7942–7949. doi: 10.1523/JNEUROSCI.1289-06.2006 16870739 PMC6674209

[5] Umilta’ MA, Berchio C, Sestito M, Freedberg D, Gallese V. Abstract art and cortical motor activation: an EEG study. Front Hum Neurosci. 2012;6. doi: 10.3389/fnhum.2012.00311 23162456 PMC3499799

[6] Sbriscia-Fioretti B, Berchio C, Freedberg D, Gallese V, Umiltà MA. ERP Modulation during Observation of Abstract Paintings by Franz Kline. PLoS One. 2013;8: e75241. doi: 10.1371/journal.pone.0075241 24130693 PMC3793982

[7] Borgomaneri S, Vitale F, Battaglia S, Avenanti A. Early Right Motor Cortex Response to Happy and Fearful Facial Expressions: A TMS Motor-Evoked Potential Study. Brain Sci. 2021;11: 1203. doi: 10.3390/brainsci11091203 34573224 PMC8471632

[8] Borgomaneri S, Gazzola V, Avenanti A. Motor mapping of implied actions during perception of emotional body language. Brain Stimul. 2012;5: 70–76. doi: 10.1016/j.brs.2012.03.011 22503473

[9] Ernst LH, Plichta MM, Lutz E, Zesewitz AK, Tupak S V., Dresler T, et al. Prefrontal activation patterns of automatic and regulated approach–avoidance reactions–A functional near-infrared spectroscopy (fNIRS) study. Cortex. 2013;49: 131–142. doi: 10.1016/j.cortex.2011.09.013 22036575

[10] Klopfer B, Kelley DM. The Rorschach Technique: A Manual for a Projective Method of Personality Diagnosis. Arch Neurol Psychiatry. 1943;49: 930. doi: 10.1001/archneurpsyc.1943.02290180154019

[11] Hermann Rorschach. Psychodiagnostik. 1921.

[12] Porcelli P, Mihura JL. Assessment of Alexithymia With the Rorschach Comprehensive System: The Rorschach Alexithymia Scale (RAS). J Pers Assess. 2010;92: 128–136. doi: 10.1080/00223890903508146 20155562

[13] Meyer GJ. Rorschach performance assessment system: somministrazione, siglatura, interpretazione e manuale tecnico. 2015.

[14] Ando A, Salatino A, Giromini L, Ricci R, Pignolo C, Cristofanelli S, et al. Embodied simulation and ambiguous stimuli: The role of the mirror neuron system. Brain Res. 2015;1629: 135–142. doi: 10.1016/j.brainres.2015.10.025 26499259

[15] Ando’ A, Pineda JA, Giromini L, Soghoyan G, QunYang, Bohm M, et al. Effects of repetitive transcranial magnetic stimulation (rTMS) on attribution of movement to ambiguous stimuli and EEG mu suppression. Brain Res. 2018;1680: 69–76. doi: 10.1016/j.brainres.2017.12.007 29247630

[16] Exner J, Exner J, Levy A, Exner J, Groth-Marnat G, Wood JM, et al. The Rorschach: A Comprehensive System. Volume 1: The Rorschach, basic foundations and principles of interpretation. 2008.

[17] Bendick MR, Klopfer WG. The Effects of Sensory Deprivation and Motor Inhibition on Rorschach Movement Responses. J Proj Tech Pers Assess. 1964;28: 261–264. doi: 10.1080/0091651X.1964.10120131 14210871

[18] FERRACUTI S. CORRELATIONS FOR THE RORSCHACH WITH THE TORRANCE TESTS OF CREATIVE THINKING. Percept Mot Skills. 1999;89: 863. doi: 10.2466/PMS.89.7.863–870

[19] Porcelli P, Meyer GJ. Construct Validity of Rorschach Variables for Alexithymia. Psychosomatics. 2002;43: 360–369. doi: 10.1176/appi.psy.43.5.360 12297604

[20] Exner JE, Erdberg Philip. The Rorschach: a comprehensive system. Volume 2, Advanced interpretation. 2005 [cited 4 Jul 2023]. Available: https://books.google.com/books/about/The_Rorschach_Advanced_Interpretation.html?hl=it&id=jrc15a9C6YUC.

[21] Giromini L, Porcelli P, Viglione DJ, Parolin L, Pineda JA. The feeling of movement: EEG evidence for mirroring activity during the observations of static, ambiguous stimuli in the Rorschach cards. Biol Psychol. 2010;85: 233–241. doi: 10.1016/j.biopsycho.2010.07.008 20654683

[22] Pineda JA. The functional significance of mu rhythms: Translating “seeing” and “hearing” into “doing.” Brain Res Rev. 2005;50: 57–68. doi: 10.1016/j.brainresrev.2005.04.005 15925412

[23] Fox NA, Bakermans-Kranenburg MJ, Yoo KH, Bowman LC, Cannon EN, Vanderwert RE, et al. Assessing human mirror activity with EEG mu rhythm: A meta-analysis. Psychol Bull. 2016;142: 291–313. doi: 10.1037/bul0000031 26689088 PMC5110123

[24] Porcelli P, Giromini L, Parolin L, Pineda JA, Viglione DJ. Mirroring Activity in the Brain and Movement Determinant in the Rorschach Test. J Pers Assess. 2013;95: 444–456. doi: 10.1080/00223891.2013.775136 23495976

[25] Pineda JA, Giromini L, Porcelli P, Parolin L, Viglione DJ. Mu suppression and human movement responses to the Rorschach test. Neuroreport. 2011;22: 223–226. doi: 10.1097/WNR.0b013e328344f45c 21346645

[26] Giromini L, Viglione DJ, Pineda JA, Porcelli P, Hubbard D, Zennaro A, et al. Human Movement Responses to the Rorschach and Mirroring Activity: An fMRI Study. Assessment. 2019;26: 56–69. doi: 10.1177/1073191117731813 28906130

[27] Oldfield RC. The assessment and analysis of handedness: the Edinburgh inventory. Neuropsychologia. 1971;9: 97–113. Available: http://www.ncbi.nlm.nih.gov/pubmed/5146491. doi: 10.1016/0028-3932(71)90067-4 5146491

[28] Rossi S, Antal A, Bestmann S, Bikson M, Brewer C, Brockmöller J, et al. Safety and recommendations for TMS use in healthy subjects and patient populations, with updates on training, ethical and regulatory issues: Expert Guidelines. Clinical Neurophysiology. 2021. pp. 269–306. doi: 10.1016/j.clinph.2020.10.003 33243615 PMC9094636

[29] Viglione DJ, Blume-Marcovici AC, Miller HL, Giromini L, Meyer G. An Inter-Rater Reliability Study for the Rorschach Performance Assessment System. J Pers Assess. 2012;94: 607–612. doi: 10.1080/00223891.2012.684118 22574907

[30] Pignolo C, Giromini L, Ando’ A, Ghirardello D, Di Girolamo M, Ales F, et al. An Interrater Reliability Study of Rorschach Performance Assessment System (R–PAS) Raw and Complexity-Adjusted Scores. J Pers Assess. 2017;99: 619–625. doi: 10.1080/00223891.2017.1296844 28375651

[31] Kivisalu TM, Lewey JH, Shaffer TW, Canfield ML. An Investigation of Interrater Reliability for the Rorschach Performance Assessment System (R–PAS) in a Nonpatient U.S. Sample. J Pers Assess. 2016;98: 382–390. doi: 10.1080/00223891.2015.1118380 26730817

[32] Giromini L, Viglione DJ, Zennaro A, Cauda F. Neural activity during production of rorschach responses: An fMRI study. Psychiatry Res Neuroimaging. 2017;262: 25–31. doi: 10.1016/j.pscychresns.2017.02.001 28208069

[33] Viglione DJ, de Ruiter C, King CM, Meyer GJ, Kivisto AJ, Rubin BA, et al. Legal Admissibility of the Rorschach and R-PAS: A Review of Research, Practice, and Case Law. J Pers Assess. 2022;104: 137–161. doi: 10.1080/00223891.2022.2028795 35180040

[34] Mihura JL, Meyer GJ, Dumitrascu N, Bombel G. The validity of individual Rorschach variables: Systematic reviews and meta-analyses of the comprehensive system. Psychol Bull. 2013;139: 548–605. doi: 10.1037/a0029406 22925137

[35] Borgomaneri S, Gazzola V, Avenanti A. Temporal dynamics of motor cortex excitability during perception of natural emotional scenes. [cited 10 Nov 2021]. doi: 10.1093/scan/nst139 23945998 PMC4187264

[36] Barchiesi G, Cattaneo L. Early and late motor responses to action observation. Soc Cogn Affect Neurosci. 2013;8: 711–719. doi: 10.1093/scan/nss049 22563004 PMC3739914

[37] Catmur C, Mars RB, Rushworth MF, Heyes C. Making Mirrors: Premotor Cortex Stimulation Enhances Mirror and Counter-mirror Motor Facilitation. J Cogn Neurosci. 2011;23: 2352–2362. doi: 10.1162/jocn.2010.21590 20946056

[38] Fossataro C, Burin D, Ronga I, Galigani M, Rossi Sebastiano A, Pia L, et al. Agent-dependent modulation of corticospinal excitability during painful transcutaneous electrical stimulation. Neuroimage. 2020;217. doi: 10.1016/j.neuroimage.2020.116897 32417451

[39] Bruno V, Fossataro C, Garbarini F. Inhibition or facilitation? Modulation of corticospinal excitability during motor imagery. Neuropsychologia. 2018;111: 360–368. doi: 10.1016/j.neuropsychologia.2018.02.020 29462639

[40] Fossataro C, Bruno V, Giurgola S, Bolognini N, Garbarini F. Losing my hand. Body ownership attenuation after virtual lesion of the primary motor cortex. European Journal of Neuroscience. 2018;48: 2272–2287. doi: 10.1111/ejn.14116 30117217

[41] Bucchioni G, Fossataro C, Cavallo A, Mouras H, Neppi-Modona M, Garbarini F. Empathy or Ownership? Evidence from Corticospinal Excitability Modulation during Pain Observation. J Cogn Neurosci. 2016;28: 1760–1771. doi: 10.1162/jocn_a_01003 27378331

[42] Di Lazzaro V, Oliviero A, Mazzone P, Insola A, Pilato F, Saturno E, et al. Comparison of descending volleys evoked by monophasic and biphasic magnetic stimulation of the motor cortex in conscious humans. Exp Brain Res. 2001;141: 121–7. doi: 10.1007/s002210100863 11685416

[43] Kammer T, Beck S, Thielscher A, Laubis-Herrmann U, Topka H. Motor thresholds in humans: a transcranial magnetic stimulation study comparing different pulse waveforms, current directions and stimulator types. Clinical Neurophysiology. 2001;112: 250–258. doi: 10.1016/s1388-2457(00)00513-7 11165526

[44] Brasil-Neto JP, Pascual-Leone A, Valls-Solé J, Cohen LG, Hallett M. Focal transcranial magnetic stimulation and response bias in a forced-choice task. J Neurol Neurosurg Psychiatry. 1992;55: 964–966. doi: 10.1136/jnnp.55.10.964 1431962 PMC1015201

[45] Rossini PM, Barker AT, Berardelli A, Caramia MD, Caruso G, Cracco RQ, et al. Non-invasive electrical and magnetic stimulation of the brain, spinal cord and roots: basic principles and procedures for routine clinical application. Report of an IFCN committee. Electroencephalogr Clin Neurophysiol. 1994;91: 79–92. doi: 10.1016/0013-4694(94)90029-9 7519144

[46] Bruno V, Fossataro C, Bolognini N, Zigiotto L, Vallar G, Berti A, et al. The role of premotor and parietal cortex during monitoring of involuntary movement: A combined TMS and tDCS study. Cortex. 2017;96: 83–94. doi: 10.1016/j.cortex.2017.09.001 28985532

[47] Burin D, Garbarini F, Bruno V, Fossataro C, Destefanis C, Berti A, et al. Movements and body ownership: Evidence from the rubber hand illusion after mechanical limb immobilization. Neuropsychologia. 2017;107: 41–47. doi: 10.1016/j.neuropsychologia.2017.11.004 29109038

[48] Fossataro C, Bucchioni G, D’Agata F, Bruno V, Morese R, Krystkowiak P, et al. Anxiety-dependent modulation of motor responses to pain expectancy. Soc Cogn Affect Neurosci. 2018;13: 1–10. doi: 10.1093/scan/nsx146 29325145 PMC5836268

[49] Garbarini F, Cecchetti L, Bruno V, Mastropasqua A, Fossataro C, Massazza G, et al. To move or not to move? Functional role of ventral premotor cortex in motor monitoring during limb immobilization. Cerebral Cortex. 2019;29: 273–282. doi: 10.1093/cercor/bhy134 29893773

[50] Dell’Anna A, Fossataro C, Burin D, Bruno V, Salatino A, Garbarini F, et al. Entrainment beyond embodiment. Neuropsychologia. 2018;119: 233–240. doi: 10.1016/j.neuropsychologia.2018.08.017 30138670

[51] Fossataro C, Bucchioni G, Bruno V, D’Agata F, Garbarini F. Pain expectancy induces freezing effects as in the actual pain: Evidence from corticospinal modulation during classical conditioning paradigm. Neuropsychological trends. 2015. p. 18: 104.

[52] West SG, Finch JF, Curran PJ. Structural equation models with nonnormal variables: Problems and remedies. [cited 4 Jul 2023]. Available: https://psycnet.apa.org/record/1995-97753-004.

[53] Osborne J. Notes on the use of data transformations. Practical Assessment, Research & Evaluation. 2002;8: 1–7. Available: http://pareonline.net/getvn.asp?v=8&n=6.

[54] Bisley JW, Goldberg ME. Attention, Intention, and Priority in the Parietal Lobe. Annu Rev Neurosci. 2010;33: 1–21. doi: 10.1146/annurev-neuro-060909-152823 20192813 PMC3683564

[55] Katsuki F, Constantinidis C. Bottom-Up and Top-Down Attention. The Neuroscientist. 2014;20: 509–521. doi: 10.1177/1073858413514136 24362813

[56] Wolfe JM. Visual search. Current Biology. 2010;20: R346–R349. doi: 10.1016/j.cub.2010.02.016 21749949 PMC5678963

[57] Serences JT, Yantis S. Selective visual attention and perceptual coherence. Trends Cogn Sci. 2006;10: 38–45. doi: 10.1016/j.tics.2005.11.008 16318922

[58] Corbetta M, Shulman GL. Control of goal-directed and stimulus-driven attention in the brain. Nat Rev Neurosci. 2002. doi: 10.1038/nrn755 11994752

[59] Vossel S, Geng JJ, Fink GR. Dorsal and Ventral Attention Systems. The Neuroscientist. 2014;20: 150–159. doi: 10.1177/1073858413494269 23835449 PMC4107817

[60] Acklin MW, Wu-Holt P. Contributions of Cognitive Science to the Rorschach Technique: Cognitive and Neuropsychological Correlates of the Response Process. J Pers Assess. 1996;67: 169–178. doi: 10.1207/s15327752jpa6701_13 16367661

[61] Vitolo E, Giromini L, Viglione DJ, Cauda F, Zennaro A. Complexity and Cognitive Engagement in the Rorschach Task: An fMRI Study. J Pers Assess. 2021;103: 634–644. doi: 10.1080/00223891.2020.1842429 33166191

[62] Ales F, Giromini L, Zennaro A. Complexity and Cognitive Engagement in the Rorschach Task: An Eye-Tracking Study. J Pers Assess. 2020;102: 538–550. doi: 10.1080/00223891.2019.1575227 30990335

[63] Heimann K, Umilta MA, Gallese V. How the motor-cortex distinguishes among letters, unknown symbols and scribbles. A high density EEG study. Neuropsychologia. 2013;51: 2833–2840. doi: 10.1016/j.neuropsychologia.2013.07.014 23911777

[64] Ticini LF, Urgesi C, Calvo-Merino B. Embodied Aesthetics: Insight from Cognitive Neuroscience of Performing Arts. 2015. pp. 103–115. doi: 10.1007/978-94-017-9379-7_7

[65] Kirsch LP, Dawson K, Cross ES. Dance experience sculpts aesthetic perception and related brain circuits. Ann N Y Acad Sci. 2015;1337: 130–139. doi: 10.1111/nyas.12634 25773627 PMC4402020

[66] Battaglia F, Lisanby SH, Freedberg D. Corticomotor Excitability during Observation and Imagination of a Work of Art. Front Hum Neurosci. 2011;5. doi: 10.3389/fnhum.2011.00079 21897813 PMC3159953

[67] Lutz A, Nassehi A, Bao Y, Pöppel E, Sztrókay A, Reiser M, et al. Neurocognitive processing of body representations in artistic and photographic images. Neuroimage. 2013;66: 288–292. doi: 10.1016/j.neuroimage.2012.10.067 23123681

[68] Naish KR, Houston-Price C, Bremner AJ, Holmes NP. Effects of action observation on corticospinal excitability: Muscle specificity, direction, and timing of the mirror response. Neuropsychologia. 2014;64C: 331–348. doi: 10.1016/j.neuropsychologia.2014.09.034 25281883

[69] Lepage J-F, Tremblay S, Théoret H. Early non-specific modulation of corticospinal excitability during action observation. European Journal of Neuroscience. 2010;31: 931–937. doi: 10.1111/j.1460-9568.2010.07121.x 20374291

[70] Ubaldi S, Barchiesi G, Cattaneo L. Bottom-Up and Top-Down Visuomotor Responses to Action Observation. Cerebral Cortex. 2015;25: 1032–1041. doi: 10.1093/cercor/bht295 24132640

[71] Romani M, Cesari P, Urgesi C, Facchini S, Aglioti SM. Motor facilitation of the human cortico-spinal system during observation of bio-mechanically impossible movements. Neuroimage. 2005;26: 755–763. doi: 10.1016/j.neuroimage.2005.02.027 15955484

[72] Finisguerra A, Amoruso L, Makris S, Urgesi C. Dissociated Representations of Deceptive Intentions and Kinematic Adaptations in the Observer’s Motor System. Cerebral Cortex. 2018;28: 33–47. doi: 10.1093/cercor/bhw346 29253254

[73] Meyer GJ, Hilsenroth MJ, Baxter D, Exner JE, Fowler JC, Piers CC, et al. An Examination of Interrater Reliability for Scoring the Rorschach Comprehensive System in Eight Data Sets. J Pers Assess. 2002;78: 219–274. doi: 10.1207/S15327752JPA7802_03 12067192

[74] Ando A, Pineda JA, Giromini L, Soghoyan G, Bohm M, Maryanovsky D, et al. Effects of repetitive transcranial magnetic stimulation (rTMS) on attribution of movement to ambiguous stimuli and EEG mu suppression. 2018 [cited 22 Mar 2018]. doi: 10.1016/j.brainres.2017.12.007 29247630

[75] Fiori F, Plow E, Rusconi ML, Cattaneo Z. Modulation of corticospinal excitability during paintings viewing: A TMS study. Neuropsychologia. 2020;149: 107664. doi: 10.1016/j.neuropsychologia.2020.107664 33130160

