# Supplementary material for: Can static Rorschach stimuli perceived as in motion affect corticospinal excitability?
Source: PLoS One. 2023 Jul 13;18(7):e0287866. doi: 10.1371/journal.pone.0287866 (PMC10343040; doi:10.1371/journal.pone.0287866)
Supplement: S1 Fig — (PDF) [file pone.0287866.s001.pdf]

Figure S-1. Standard R-PAS administration procedure

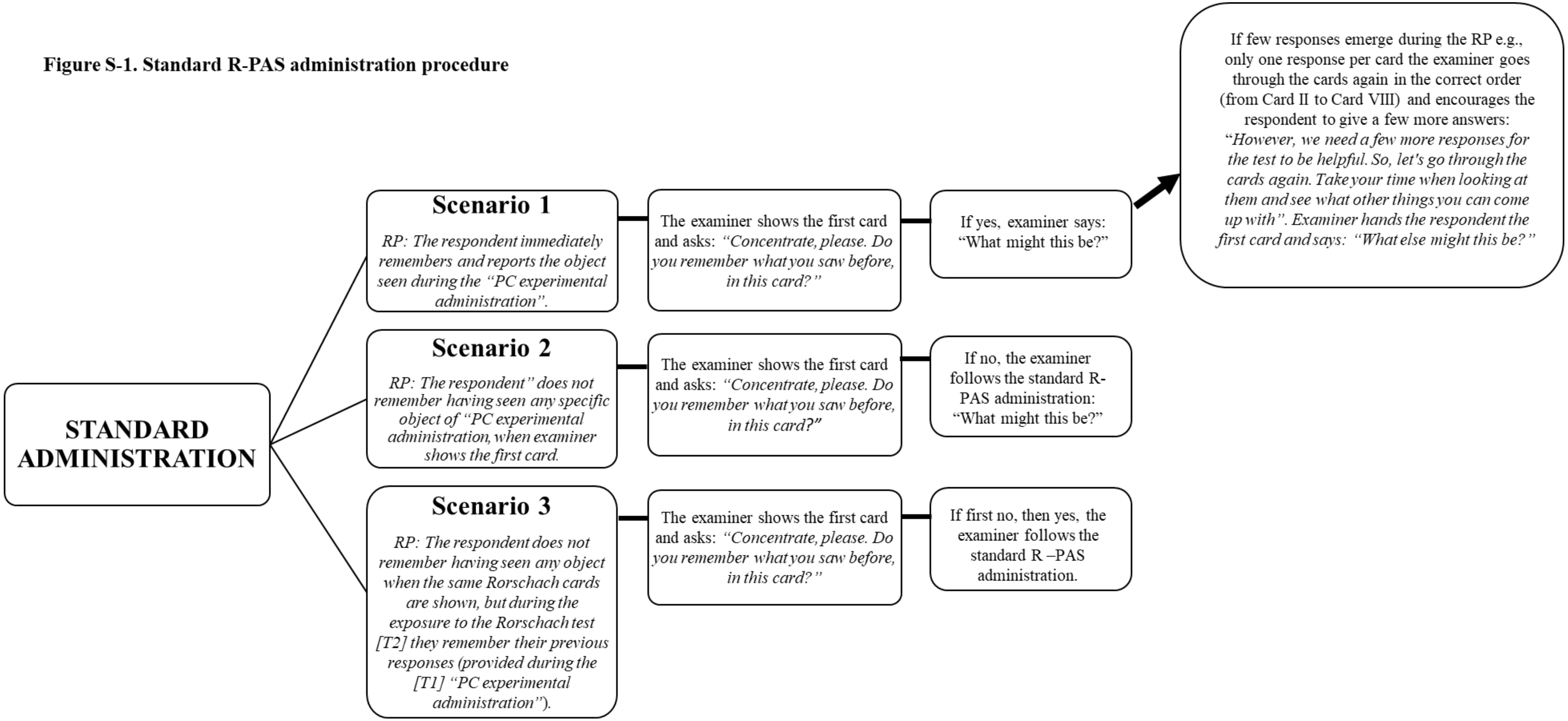

Additional Information: During the "PC administration", a total of 30 stimuli were presented in a pseudorandom order using the E-prime presentation software: 15 of them were M cards (5 II, 5 III, and 5 VII) and the other 15 were Non-M Cards (5 V, 5 VI, and 5 VIII). Thus, a Rorschach card was seen 5 times and the participant could have possibly perceived movement and even feeling of 5 percepts (in movement) per card. Therefore, we thought of the possibility of a first scenario in which the respondent immediately could have remembered and reported the object seen during the "experimental administration": so that the participant would have to provide a) the same number of responses given during the experimental administration and, more importantly, b) responses potentially remembered.
