# Supplementary figures and images for: Can static Rorschach stimuli perceived as in motion affect corticospinal excitability?
Source: PLoS One. 2023 Jul 13;18(7):e0287866. doi: 10.1371/journal.pone.0287866 (PMC10343040; doi:10.1371/journal.pone.0287866)

Figure S-2. Correlation Graph

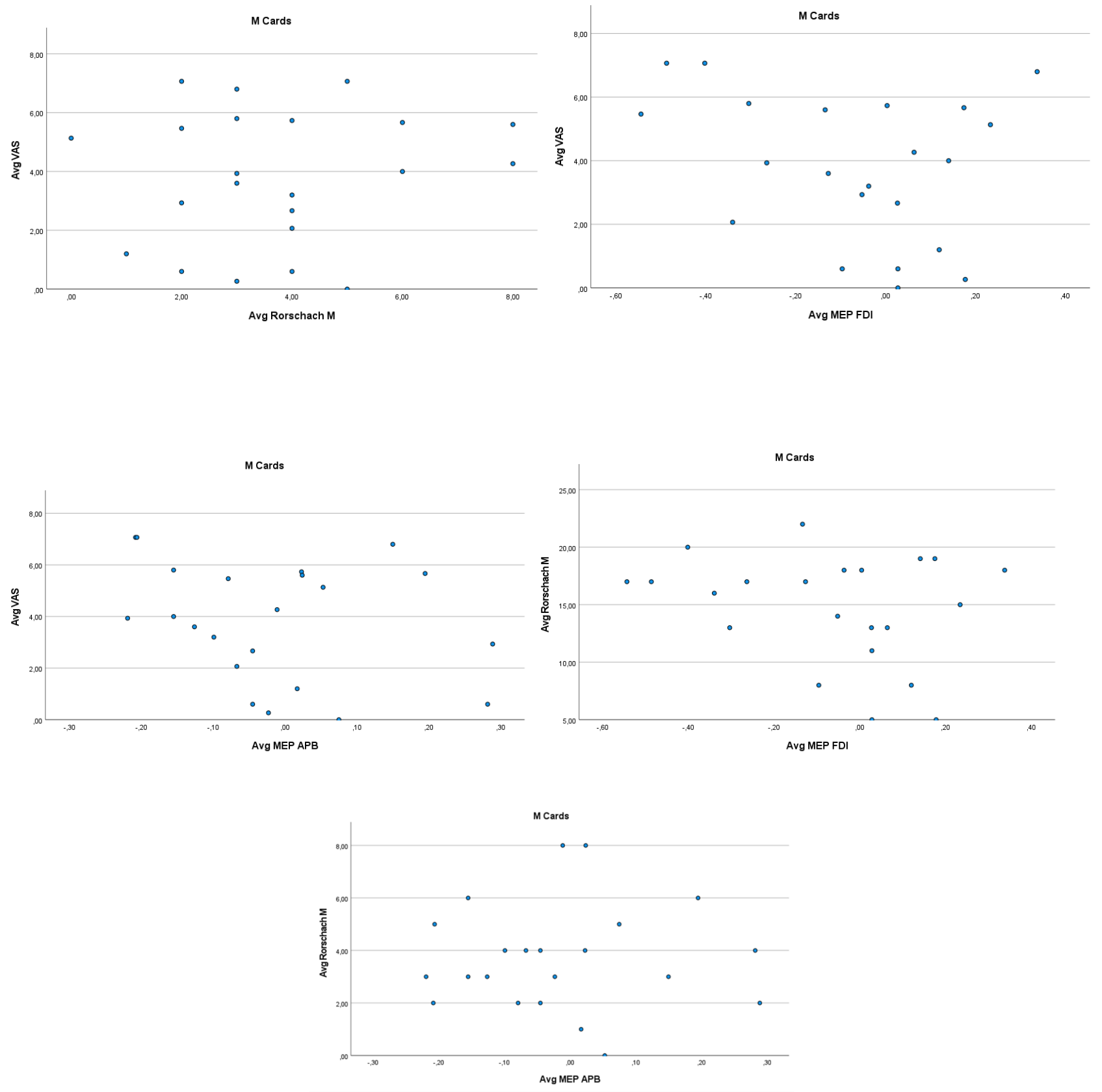

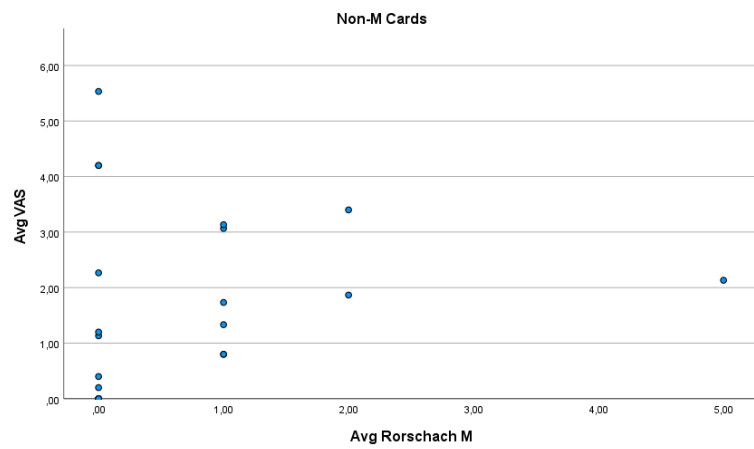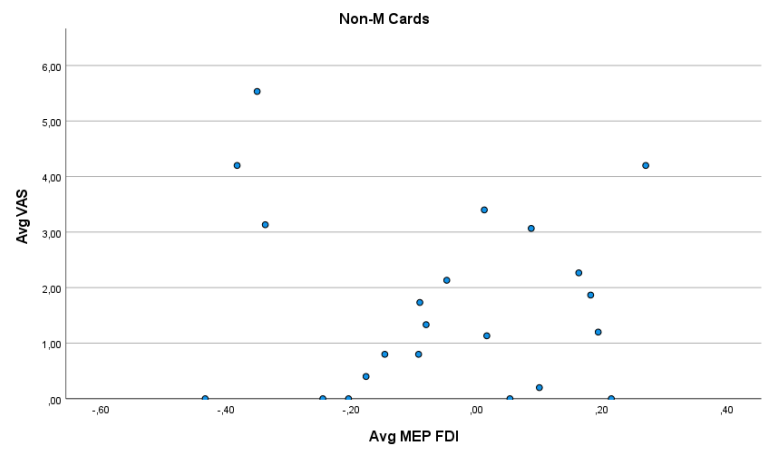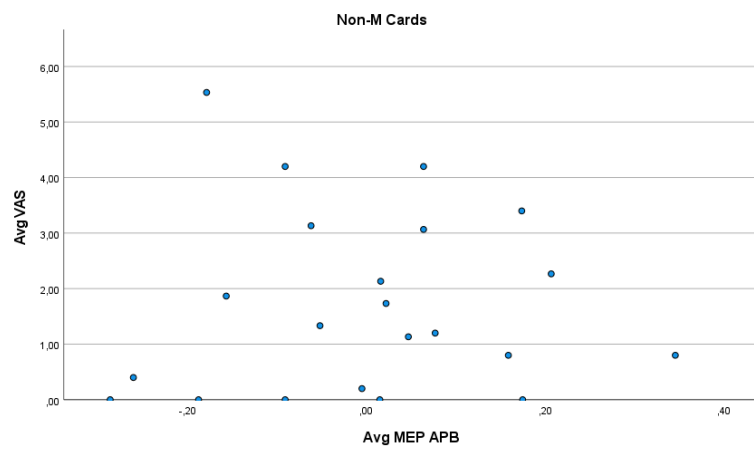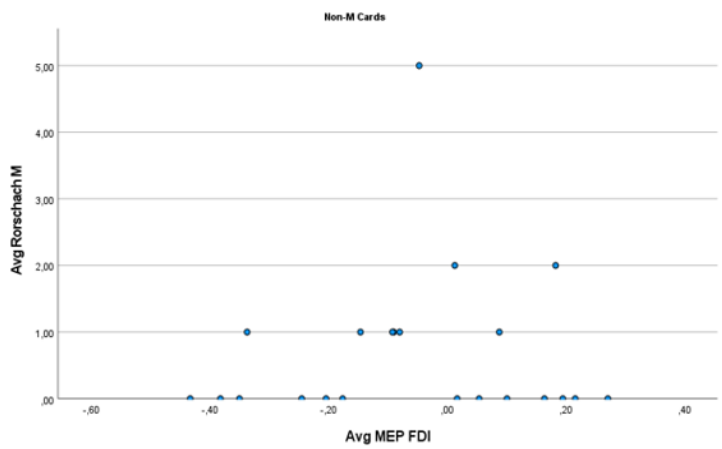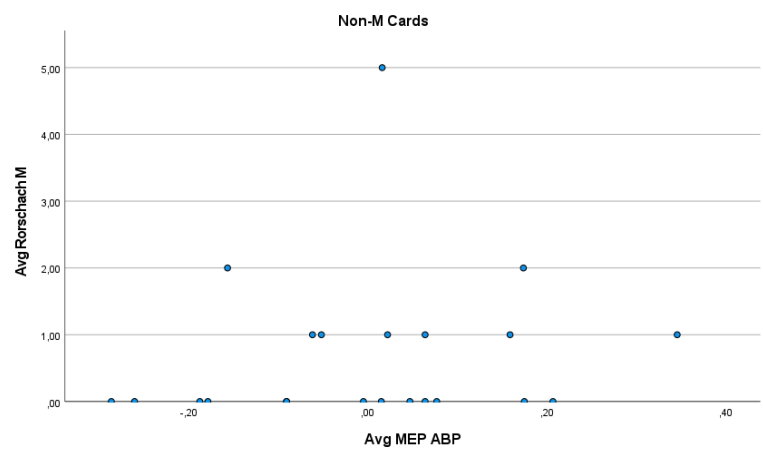

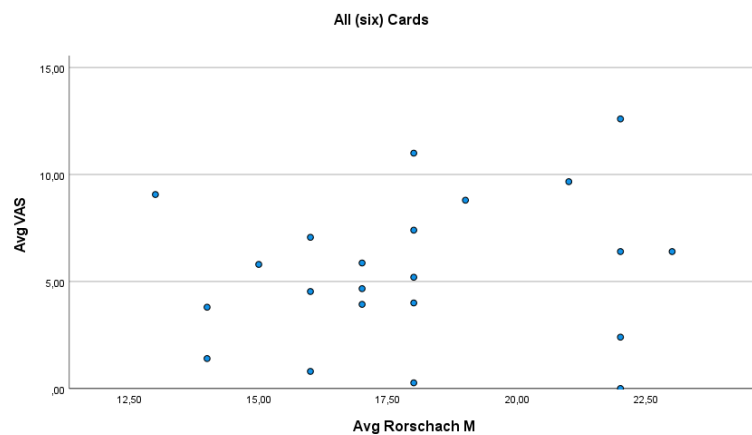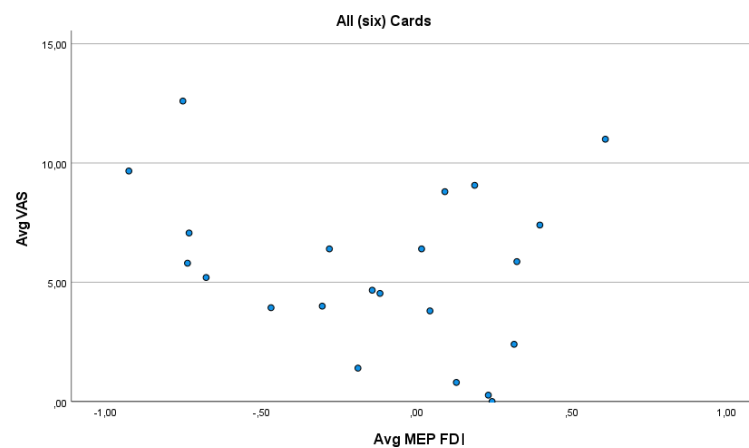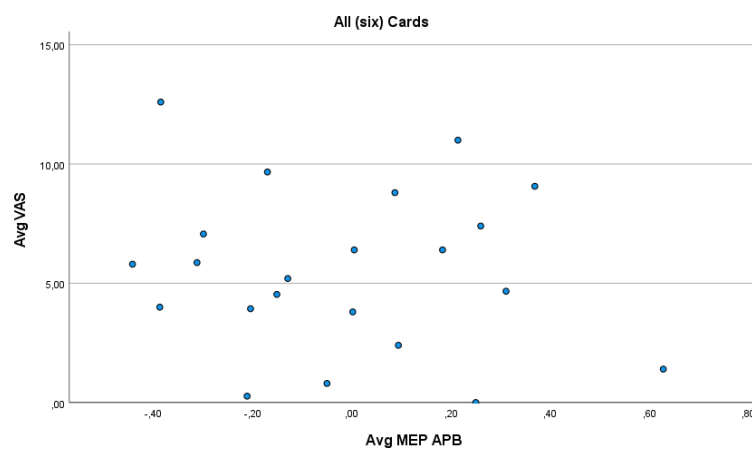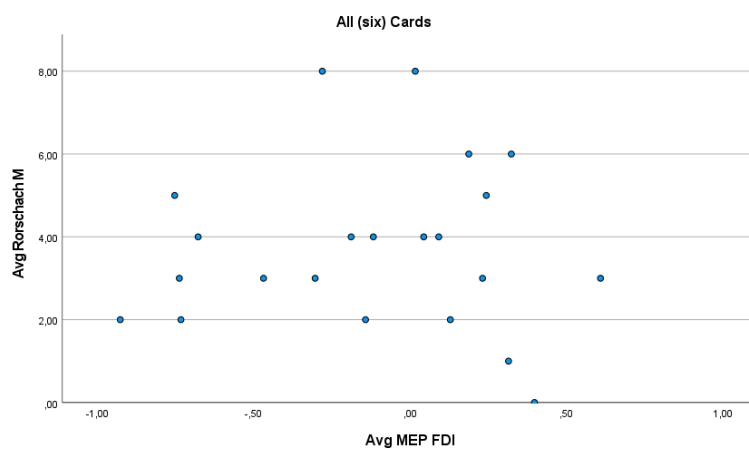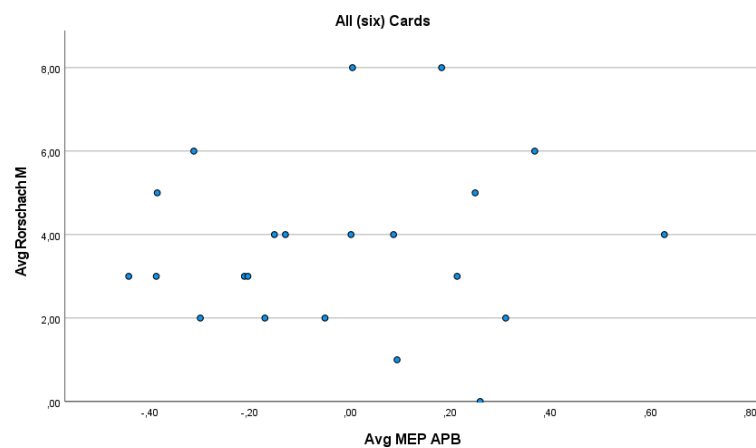

Supplement: S2 Fig — (PDF) [file pone.0287866.s002.pdf]
